# Monitoring IgG against *Mycobacterium tuberculosis* proteins in an Asian elephant cured of tuberculosis that developed from long-term latency

**DOI:** 10.1038/s41598-022-08228-7

**Published:** 2022-03-12

**Authors:** Satoshi Ishikawa, Yuriko Ozeki, Satomi Suga, Yasuhiko Mukai, Haruka Kobayashi, Erina Inouchi, Shaban A. Kaboso, Gebremichal Gebretsadik, Desak Nyoman Surya Suameitria Dewi, Akihito Nishiyama, Yoshitaka Tateishi, Hayato Takihara, Shujiro Okuda, Shiomi Yoshida, Naoaki Misawa, Sohkichi Matsumoto

**Affiliations:** 1 Department of Bacteriology, Niigata University School of Medicine, 1-757, Asahimachi-Dori, Chuo-ku, Niigata, Niigata 951-9510 Japan; 2 Fukuyama Zoo, 276-1, Fukuda, Ashida-cho, Fukuyama, Hiroshima 720-1264 Japan; 3 Department of Microbiology, Faculty of Medicine, Universitas Ciputra, CitraLand CBD Boulevard, Made, Sambikerep, Surabaya, 60219 Indonesia; 4 Division of Bioinformatics, Niigata University Graduate School of Medical and Dental Sciences, 2-5274, Gakkocho-dori, Chuo-ku, Niigata, Niigata 951-8514 Japan; 5 Medical AI Center, Niigata University School of Medicine, 2-5274, Gakkocho-dori, Chuo-ku, Niigata, Niigata 951-8514 Japan; 6 Nagasaki University Graduate School of Biomedical Sciences, 1-12-4 Sakamoto, Nagasaki, 852-8523 Japan; 7 National Hospital Organization Kinki-Chuo Chest Medical Center Clinical Research Center, 1180 Nagasone-cho, Kita-ku, Sakai, Osaka 591-8555 Japan; 8 Graduate School of Medicine and Veterinary Medicine, University of Miyazaki, 5200, Kihara, Kiyotake-cho, Miyazaki, Miyazaki 889-1692 Japan; 9 Laboratory of Tuberculosis, Institute of Tropical Disease, Universitas Airlangga, Kampus C Jl. Mulyorejo, Surabaya, 60115 Indonesia

**Keywords:** Tuberculosis, Immunology, Diagnosis

## Abstract

Tuberculosis (TB) is fatal in elephants, hence protecting elephants from TB is key not only in the conservation of this endangered animal, but also to prevent TB transmission from elephants to humans. Most human TB cases arise from long-term asymptomatic infections. Significant diagnostic challenges remain in the detection of both infection and disease development from latency in elephants due to their huge bodies. In this study, we assessed cryopreserved sera collected for over 16 years, from the first Japanese treatment case of elephant TB. Semi-quantification of IgG levels to 11 proteins showed high detection levels of 3 proteins, namely ESAT6/CFP10, MPB83 and Ag85B. The level of IgG specific to these 3 antigens was measured longitudinally, revealing high and stable ESAT6/CFP10 IgG levels regardless of onset or treatment. Ag85B-specifc IgG levels were largely responsive to onset or treatment, while those of MPB83 showed intermediate responses. These results suggest that ESAT6/CFP10 is immunodominant in both asymptomatic and symptomatic phases, making it useful in the detection of infection. On the other hand, Ag85B has the potential to be a marker for the prediction of disease onset and in the evaluation of treatment effectiveness in elephants.

## Introduction

Tuberculosis (TB) is an infectious disease caused by *Mycobacterium tuberculosis* (*Mtb*); the leading cause of human death due to a single pathogen before the emergence of SARS-CoV-2^1^. *Mtb* infects a wide variety of mammalian species^2–4^, including elephants (*Elephas maximus and Loxodonta africana*) with cases being reported worldwide, in captivity as well as in the wild^5^. TB is fatal in elephants, hence protecting them from this disease is key in the conservation of this endangered animal^6^. In addition, cross-species transmission between elephants and other mammals in the zoo^7,8^, or between elephants and humans, such as keepers or handlers^8–11^, has been reported. Therefore, elephant TB is a serious infectious disease and a public health concern.

In humans, most symptomatic TB cases develop from long-term asymptomatic infection known as latent TB. Latent TB patients are at risk of developing TB and transmitting *Mtb* bacteria in future^12^. There is little debate about whether such latent TB occurs in elephants, although some reports implicate the presence of asymptomatic infection in elephants^13,14^. The current gold standard for TB testing in elephants is the trunk wash culture and its PCR^15^, but these methods do not detect *Mtb* infection without bacterial discharge. Therefore, when the trunk wash test comes out positive, the elephant has already shed bacteria, and might have infected other elephants, humans or distinct species nearby. To prevent transmission, it is thus important to detect *Mtb* infection before the start of bacterial discharge, which necessitates the need for pre-shedding diagnostic methods.

Latent TB in humans can only be detected by immunological tests^12^, which have also been tried in elephants. Previous studies have shown that the tuberculin skin test is not sufficiently sensitive in elephants^16^. Interferon-gamma release assay (IGRA), a recent standard test in humans, has not yet been practical in elephants although there have been some attempts to develop it^17–19^. A rapid immuno-test kit, Dual Path Platform (DPP) VetTB assay for elephants (Chembio Diagnostic Systems, Medford, NY), is used worldwide for elephant TB screening^20,21^. DPP can detect antibodies against ESAT6/CFP10 (a fusion protein with 6 kDa early secretory antigenic target) and MPB83 (a 10 kDa culture filtrate protein)^20^. However, given that *Mtb* produces many antigens, we considered the possible existence of more suitable antigens for TB testing other than MPB83 and ESAT6/CFP10.

Imaging tests such as X-rays and CT play an important role in assessing the progress of TB in humans^22^. However, chest imaging in elephants is impractical due to their large bodies, which limits the applicability of available monitoring methods. Therefore, new elephant specific methods are required for early TB detection, development prediction and transmission prevention. Studies have shown that antibody levels to certain *Mtb* antigens increase with the progression of TB in elephants^23,24^. Hence longitudinal and quantitative antibody testing is a potentially new monitoring method for TB in elephants. However, it is difficult to validate or establish such tests because of the rarity of pre-TB sera collection in elephants.

In 2016, an Asian elephant was diagnosed with symptomatic TB at the Fukuyama Zoo in Japan, and *Mycobacterium caprae* was later isolated^25,26^—*Mtb* complex species including *M. caprae* were reclassified as *Mtb* in 2018^27^. Prior to this, only a few cases of TB in elephants were reported in Japan, and all of them were found at postmortem necropsy^4,28–30^. The Fukuyama Zoo elephant with symptomatic TB, herein referred to as the TB positive elephant, completed its TB treatment in 2018^25^. In this study, we investigated the presence of *Mtb* antigen-specific IgG in the sera of the TB positive elephant, and searched for new markers to detect *Mtb* infection. Moreover, since the sera had been cryopreserved for a total of 16 years, we analyzed the changes in IgG levels longitudinally from 12 years before TB onset to 1 year after treatment completion.

## Results

### Rapid serological test assays

**Figure 1:**
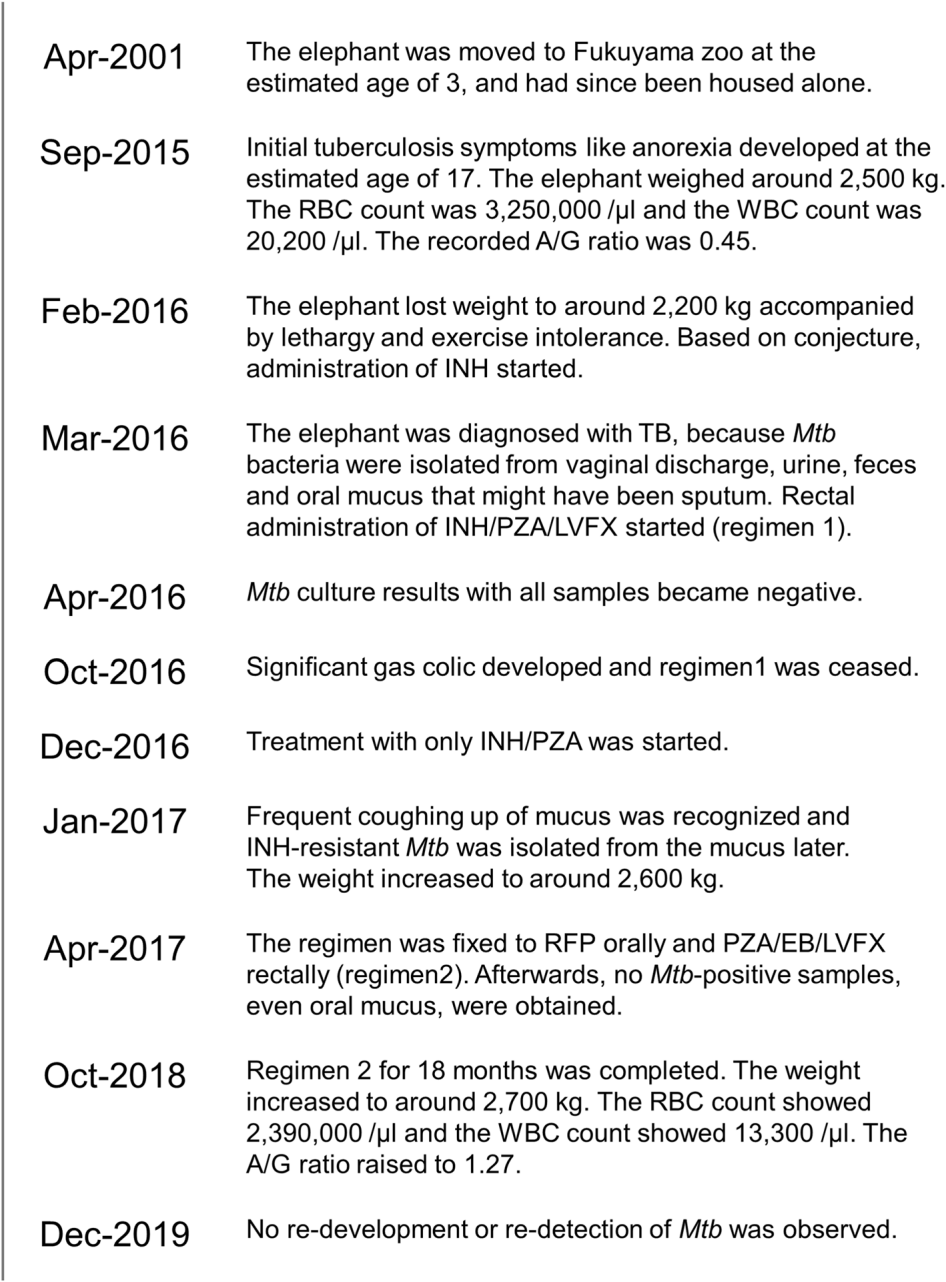
History of the TB positive elephant. This clinical information was added based on the report by Suga et al.^25^. INH, PZA, LVFX, RFP, and EB in the figure are abbreviations for isoniazid, pyrazinamide, levofloxacin, rifampicin, and ethambutol. RBC and WBC are also abbreviations for red blood cell and white blood cell. The A/G ratio is the ratio of serum albumin divided by the globulin concentration.

We obtained sera from the TB positive elephant at 53 time points from November 2003 (approximately 12 years before TB diagnosis), until December 2019 (13 months after the completion of TB treatment). The history of this elephant is briefly described in Fig. 1. We also obtained sera from 7 healthy Asian elephants kept in Japan as negative control.

**Figure 2:**
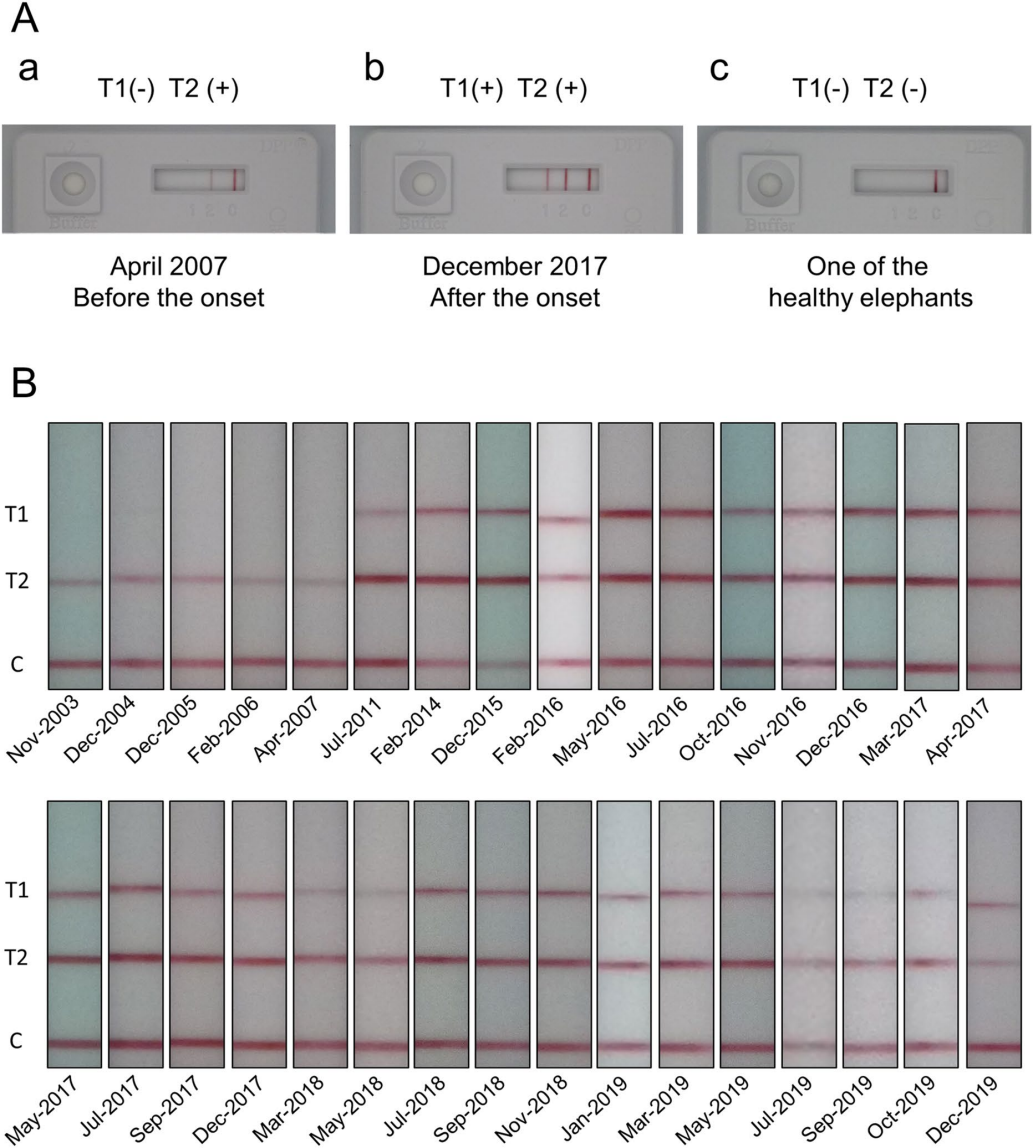
Antibody detection by DPP VetTB assay. (**A**) These 3 images are representative DPP results in the TB positive elephant (**a** and **b**) and the elephants with no TB symptoms (**c**). In the test windows, the appearance of the red line on the left (T1 line) and the red line in the middle (T2 line) indicate the presence of MPB83 and ESAT6/CFP10 antibodies, respectively. The red line on the right is the control: if it does not appear, the test is invalid. (**B**) The results of DPP VetTB assays in the TB positive elephant, from November 2003 until December 2019 were captured on a compact digital camera. Only the area around T1, T2, and control lines were cropped from the images, rotated and arranged in chronological order. The uncropped images of all DPP results in Fig. 2 are presented in Supplemental Fig. 2.

We first conducted DPP and tentatively checked the presence of ESAT6/CFP10 and MPB83 antibodies (Fig. 2A and B). We tested sera collected at 32 different time points from the TB positive elephant, and each possessed ESAT6/CFP10 antibodies (Fig. 2A-a, b and B). On the other hand, MPB83 antibodies were not detected from November 2003 to April 2007 (Fig. 2A-a, and B), but were first observed in July 2011—more than 4 years before TB diagnosis—and were continuously present through to December 2019 (Fig. 2B). In contrast, all the 7 sera from healthy elephants did not show any reaction to both ESAT6/CFP10 and MPB83 (Fig. 2A-c). According to DPP instructions, sera from November 2003 to April 2007 were suggestive of TB or mycobacteriosis, while sera from July 2011 to December 2019 were suggestive of TB. On the other hand, the 7 sera from healthy elephants were nonreactive.

### An immunodominant protein other than ESAT6/CFP10 and MPB83 in the TB positive elephant

In order to quantitatively monitor ESAT6/CFP10 and MPB83-specific IgG levels and also find other antigens appropriate for TB testing in elephants, *Mtb* protein antigens were prepared and antigen-specific IgG levels were measured by quantitative enzyme-linked immune sorbent assay (ELISA).

We prepared the following ESAT6/CFP10 like *Mtb*-specific antigens: Rv2074, Rv2652c, Rv3878, Rv1507A, and Rv3108. We also prepared MPB83 like immunodominant antigens in mycobacteria such as, antigen 85B (Ag85B), heparin-binding hemagglutinin (HBHA), alpha-crystallin like protein (Acr), and mycobacterial DNA-binding protein 1 (MDP1). Purified protein derivative (PPD) was used as positive control. Bioinformatics analysis of the genome suggested that 10 of the 12 tested proteins were encoded in the *Mtb* variant *caprae* strains that had infected the elephant. However, genes encoding homologues of Rv2074 and Rc2652c were not observed.

**Figure 3:**
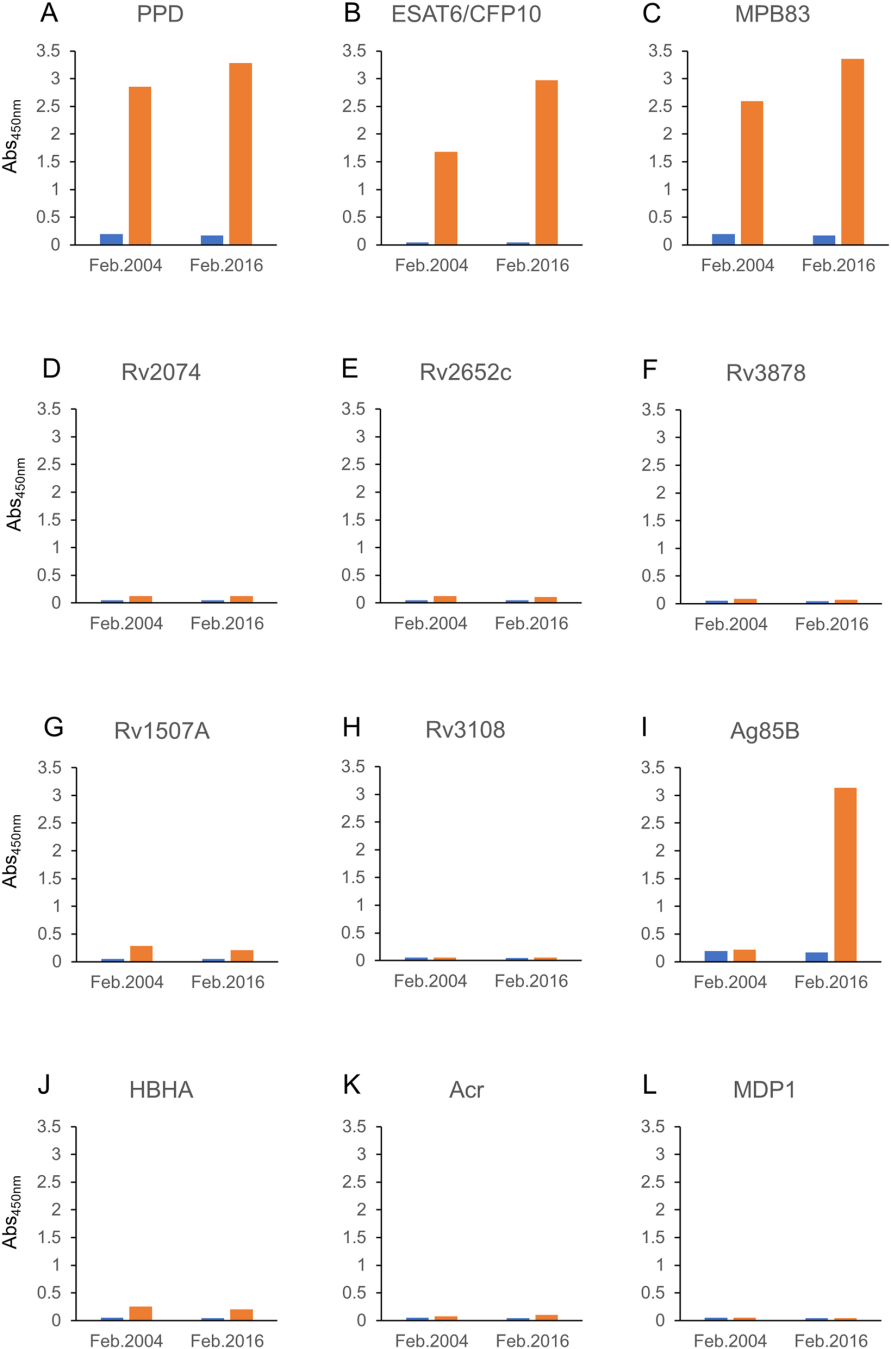
*Mtb* antigen-specific IgG levels before and after the onset of TB. The level of IgG against 11 different *Mtb* antigens in the sera from the TB positive elephant were measured with ELISA (**A** to **L**). The sera were bled in February 2004, when the elephant showed no symptoms, and in February 2016, when it showed severe TB symptoms but had not received treatment. The blue bars represent control values measured with PBS without antigen. The orange bars represent the test values. The vertical lines show Abs values measured at OD 450 nm.

For the pilot study, we used sera bled in February 2004 and February 2016, when the elephant had no symptoms and when it had severe symptoms with no TB treatment, respectively. ELISA data showed that both February 2004 and February 2016 sera showed elevated levels of PPD, ESAT6/CFP10 and MPB83-specific IgG antibodies (Fig. 3A, B and C). Regarding Ag85B, the February 2004 serum did not show any reaction. However, high IgG titer was observed in the February 2016 serum (Fig. 3I). As expected, we could not detect IgG antibodies against Rv2074 and Rc2652c (Fig. 3D, E), neither could we find IgG against the other 6 tested proteins, namely Rv3878, Rv2074, Rc2652c HBHA, Acr, and MDP1 despite the presence of their genes in the isolated *Mtb* var. *caprae* (Fig. 3F, G, H, J, K and L).

**Figure 4:**
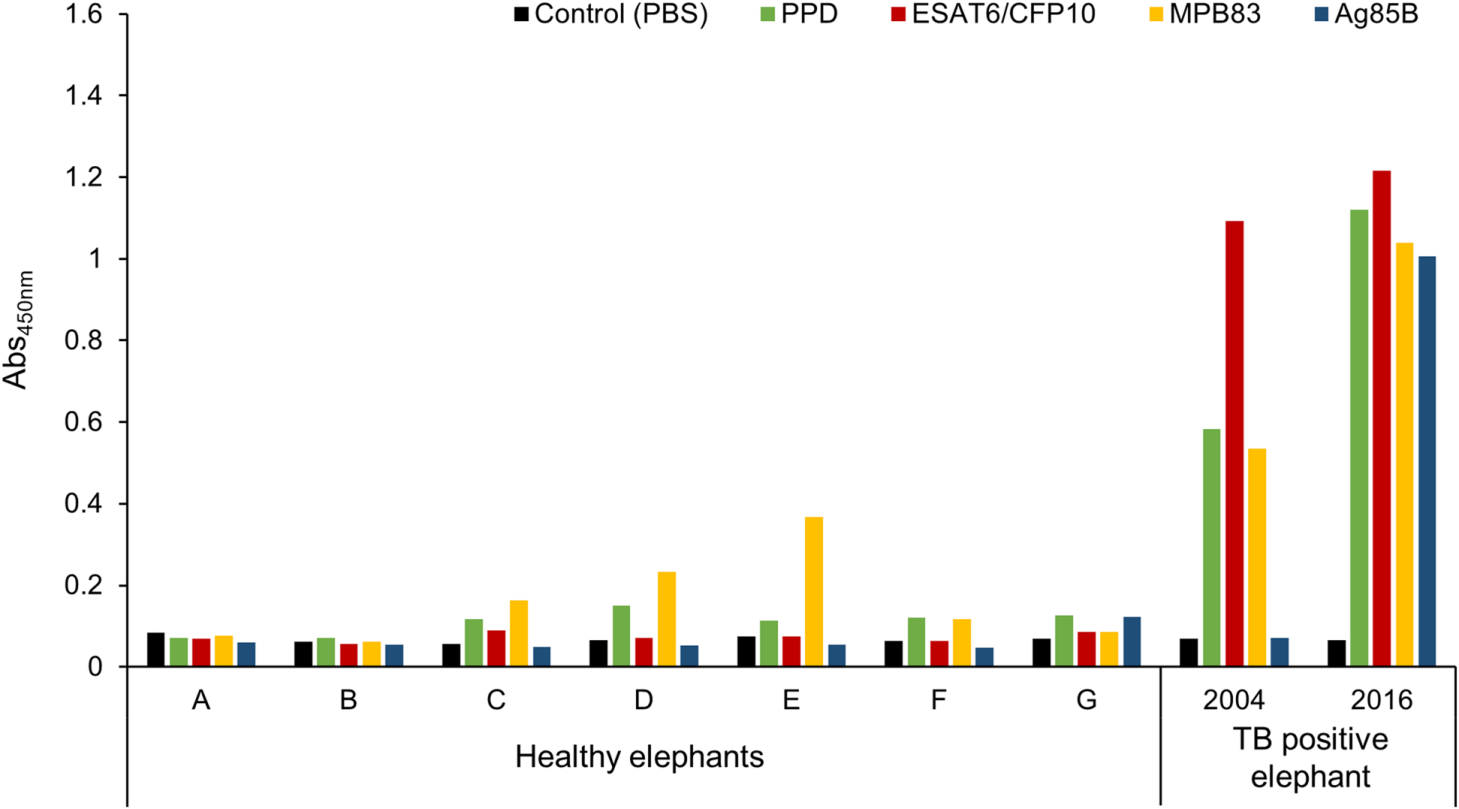
*Mtb* antigen-specific IgG levels in healthy and TB positive elephants. The IgG levels to PPD and 3 *Mtb* antigens—PPD (green bars), ESAT6/CFP10 (red bars), MPB83 (yellow bars) and A85B (blue bars)—in the sera from 7 healthy elephants (**A** to **G**) were measured with ELISA. They were compared with the IgG values of the TB positive elephant sera from February 2004 and February 2016. The black bars represent the values measured for the control with PBS without antigen. The vertical line shows Abs value measured at OD 450 nm.

To validate antibody specificity, we also tested IgG levels to PPD and 3 *Mtb* antigens—ESAT6/CFP10, MPB83 and Ag85B—in sera derived from the 7 healthy elephants (Fig. 4). According to Tukey honestly significant difference test, ESAT6/CFP10 and Ag85B absorbance (Abs) were not significantly different from the phosphate-buffered saline (PBS) control (*p* = 1.000). This implies that ESAT6/CFP10 and Ag85B-specific IgG were not detected in the healthy elephants, suggesting their specificity for elephant TB. On the other hand, even though these 7 sera did not respond to DPP, several elephants showed presence of MPB83-specific IgG, although not as high as the TB positive elephant (Fig. 4). Statistics on the 7 elephants detected a significant deference between MPB83 and PBS control Abs (*p* = 0.024), suggesting IgG non-specificity against MPB83 in the diagnosis of *Mtb* infection in elephants.

### Longitudinal evaluation of PPD, ESAT6/CFP10, MPB83, and Ag85B-specific IgG levels

**Figure 5:**
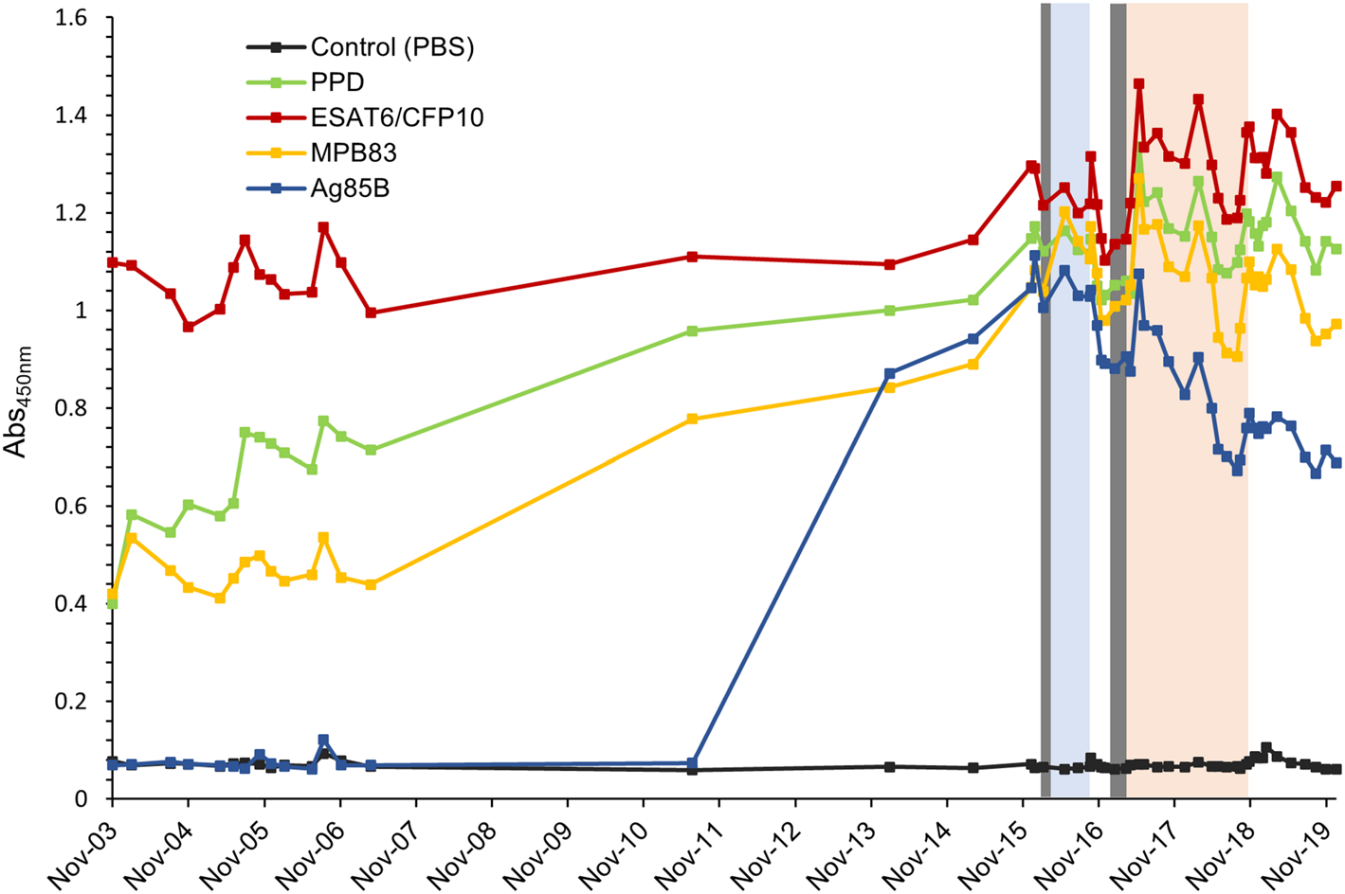
Longitudinal changes of *Mtb* antigen-specific IgG levels in the TB positive elephant. The IgG levels to PPD and 3 *Mtb* antigens—PPD (green line), ESAT6/CFP10 (red line), MPB83 (yellow line) and Ag85B (blue line)—in 53 sera from the TB positive elephant, collected 12 years before diagnosis until 13 months after treatment completion, were measured longitudinally with ELISA. The black line represents changes in control values measured with PBS without antigen. The vertical line shows Abs value measured at OD 450 nm. The period when *Mtb* was detected by culture or PCR from oral mucus or feces is shown in gray. Treatment with regimen 1 (isoniazid, pyrazinamide and levofloxacin) was carried out during the period shown in light blue, and treatment with regimen 2 (rifampicin, ethambutol, pyrazinamide and levofloxacin) was carried out during the period shown in light red.

In order to assess the kinetics of *Mtb* antigen-specific IgG levels along with latency, onset of TB, and drug treatment, the level of IgG against PPD and the 3 *Mtb* antigens—ESAT6/CFP10, MPB83 and Ag85B—were monitored longitudinally in the TB positive elephant (Fig. 5).

Regarding PPD, the IgG level increased 2.8 times from November 2003 when the elephant had no TB symptoms, to February 2016 when the elephant showed severe symptoms. The highest value was observed in May 2017 during treatment, and was sustained until after completion of treatment.

Anti-ESAT6/CFP10 IgG level was already high in November 2003, and further increased 1.1 times in February 2016. The said IgG level was highest in May 2017 during treatment and remained consistently high even after the treatment was over.

Anti-MPB83 IgG level increased 2.5 times from November 2003 to February 2016. The value reached the highest in May 2017 and then slightly declined with treatment. It decreased 0.9 times from the treatment initiation (February 2016) to the end of the study (December 2019).

From November 2003 to July 2011, there was no significant difference in the Abs between Ag85B and PBS control following comparison with Wilcoxon signed-rank test (*p* = 0.706), suggesting that the production of IgG against Ag85B was almost absent in this period. However, the anti-Ag85B IgG level dramatically increased 11.8 times in February 2014—almost 2 years before TB onset—and later reached the highest in January 2016. At treatment initiation (February 2016), the IgG level was 14.4 times higher than that of November 2003, which then decreased with treatment (0.7 times from February 2016 to December 2019).

Consequently, Anti-ESAT6/CFP10 IgG levels were high and stable regardless of onset or treatment. On the other hand, anti-Ag85B IgG levels were largely responsive to onset or treatment, while those of PPD and MPB83 showed intermediate responses.

## Discussion

In order to contribute to the development of elephant TB testing, we examined serum levels of IgG against 11 antigens from the first elephant to receive TB treatment in Japan. Among the tested antigens, ESAT6/CFP10 maintained high IgG levels regardless of onset or treatment. Moguche et al*.* reported that ESAT6 is expressed throughout the infection period, including the chronic phase in mice and human infected with *Mtb*^31^, which is similarly suggested by our present results in elephants. The specificity of Anti-ESAT6/CFP10 IgG was implied by the negative results obtained from the 7 healthy elephants relative to the *Mtb*-infected one. ESAT6 and CFP10 encoded by the RD1 gene are secreted proteins that promote the transfer of *Mtb* into the host cell cytoplasm^32^ and inhibit the function of TLR2, a major host sensor against *Mtb*^33^. These antigens are present in *Mtb*, but are absent in the attenuated vaccine strain, BCG^34^ and most non-tuberculous mycobacteria^35^. In addition, IGRA which detects T cell responses to ESAT6/CFP10^36^ is widely used for the diagnosis of latent TB in humans because of its bioactivity that strongly induces IFN-gamma production from effector T cells sensitized to these antigens^37^. Similarly, some studies have reported high sensitivity and specificity of ESAT6/CFP10 antibodies in elephants^20,23,38^. Our study results support these reports, and reiterates the usefulness of ESAT6/CFP10 immune response testing for the detection of *Mtb* infection in elephants.

In contrast, anti-Ag85B IgG levels changed according to the disease status. Majority of the studies conducted on Ag85B were done in humans and mice^31,39–42^, but very few on elephants. Ag85B is a mycolyltransferase secreted by mycobacteria and is involved in the final stage of mycobacterial cell wall assembly by transferring mycolic acid to sugars like trehalose^39^. Therefore, the secretion of Ag85B is thought to increase with the activation of *Mtb* replication. In fact, Moguche et al*.* pointed out that the expression of Ag85B is low in the chronic stage of *Mtb* infection, when the disease has not yet developed, in mice and humans^31^. Furthermore, Lee et al*.* reported that anti-Ag85B IgG levels in humans are significantly higher in active TB than in latent TB, which facilitates the distinction between the two states^42^. The results in the present study, which is the first report to quantitatively monitor anti-Ag85B IgG levels in a TB positive elephant, suggest that TB development increases anti-Ag85B IgG levels in *Mtb*-infected elephants, which is similar in humans. Furthermore, we found that these levels gradually decreased with treatment, and the decreasing rate was the highest among the tested antigens. These results suggest that anti-Ag85B IgG levels can be used to evaluate both TB progression and treatment efficacy in elephants.

MPB83 antibodies were not detected by DPP from November 2003 to April 2007, whereas IgG was detected by ELISA (Fig. 5). This could be due to differences in the detection limit of the techniques. DPP results turned positive in July 2011 (Fig. 2B), while ELISA showed a sharp increase of Abs at the same time (Fig. 5). These changes occurred more than 4 years before diagnosis, and preceded the detection of IgG against Ag85B.

Regarding the present results, ESAT6/CFP10 antibodies were detected by both DPP and ELISA in November 2003. These results suggest that the elephant had been infected with *Mtb* for more than 12 years prior to diagnosis. In contrast, IgG against Ag85B was absent during the initial 7 years and 9 months. In human latent TB, *Mtb* is dormant and it is characterized by low metabolic activity, inability to replicate in culture, and high resistance to external bactericidal factors^43^. The lack of anti-Ag85B IgG, which is produced during active *Mtb* replication, suggests that *Mtb* in the elephant was dormant for more than 7 years and 9 months, and that the elephant was in a state that can be called latent TB.

Detecting antibodies is likely to be beneficial for the diagnosis of TB in elephants, as recent literature has shown its usefulness and recommended its use^15,21^. Furthermore, antibody levels in human TB patients have been found to correlate with bacterial load^44^, while several publications on elephants have reported that the fluctuation in antibody levels is associated with disease onset or treatment^23,24^. The present study also suggests that the disease status affected IgG levels in the TB positive elephant. Therefore, while it is difficult to collect sputum and take chest X-rays, monitoring IgG levels in *Mtb*-infected elephants may be useful. In particular, Ag85B showed a large discrepancy in IgG levels between the disease onset and during treatment. As such, it is expected to be a new monitoring marker to predict the disease onset, which is useful for early and appropriate treatment, as well as to evaluate the treatment efficacy. However, since this study was conducted on a single case, further validation with a larger number of elephants will be necessary. On the other hand, as there are few reports on successful TB treatment in elephants, the results obtained in this study will be useful for the future development of elephant TB medicine.

## Methods

### Serum specimens from elephants

We tested sera obtained at 53 different time points from a TB positive elephant, that were sampled from 2003 to 2019 (estimated age of 3 to 20 years old), and 7 sera from 7 other Asian elephants in Japan with no suspected TB symptoms (healthy elephants). All sera were sampled for regular medical health check.

Blood was collected from auricular veins, incubated over 30 min at room temperature for clotting, and sera obtained by centrifugation at 1,500×*g* for 5 to 10 min to remove the clots. The sera were then kept in a freezer at − 20 °C until analysis.

### Dual path platform VetTB assay

DPP assay was applied for analysis of serum samples. DPP detects antibodies against ESAT6/CFP10 and MPB83 with protein A/G^20^. Assays were performed according to the manufacturer’s protocol. The results were confirmed with the naked eye and photographed with a digital camera.

### Searching for antigen genes in the isolated *Mtb* strain

Using the Burrows-Wheeler Aligner^45^, whole genome sequence files of *Mtb* variant *caprae* DRR120408 and DRR120409, isolated from the TB positive elephant^26^ were mapped to the *Mtb* (H37Rv) genome. The presence or absence of 12 antigen genes—ESAT6 (Rv3875), CFP10 (Rv3874), MPB83 (Rv2873), Rv2074, Rv2652c, Rv3878, Rv1507A, Rv3108, Ag85B (Rv1886c), HBHA (Rv0475), Acr (Rv2031c) and MDP1 (Rv2986c)—was analyzed with featureCounts^46^.

### Construction and purification of ELISA antigens

The 11 antigens used for ELISA were prepared as follows. PPD prepared from *Mtb* strain Aoyama B was purchased from Japan BCG Laboratory (Tokyo, Japan). Recombinant ESAT6/CFP10, HBHA, Acr and MDP1 were obtained as described previously^47,48^. Ag85B was purified from *Mtb* H37Rv culture filtrate by the method previously reported^49^.

For expression of Rv2074, Rv2652c, Rv3878, Rv1507A, and Rv3108 as 6XHistidine tagged proteins, each gene was amplified by polymerase chain reaction (PCR) with the following synthesized primers: CCCCATATGgcgatggtcaacaccactac and CCCAAGCTTggcccggtcgagcagatccgcgg for Rv2074 (420 bp), CCCATAtgccatcgccagcaaccgccc and CCCAAGCTTccggtctggggcgaacgggttga for Rv2652c (633 bp), CCCATatggctgaaccgttggccgtcgatcc and CCAAGCTTcaacgttgtggttgttgaggg for Rv3878 (849 bp), CCCATatgcaatcaggtcaaaatatcctcg and CCAAGCTTacccgctagaaggccggtgac for Rv1507A (510 bp), CCCATatgacacccaatgcggcgagtaccgg and AAGCTTgggcggaatccgaccactcatg for Rv3108 (447 bp) targeting the *Mtb* H37Rv genomic DNA as a template. Each DNA fragment was digested with Nde1 and HindIII and ligated into the same site in pET22b (Novagen, Madison, WI). pET22b containing each gene with 6XHistidine was transformed into ClearColi BL21 (DE3) (Lucigen, Middleton, WI) according to the manufacturer’s instructions. Bacteria were cultured in LB broth containing carbenicillin (50 μg/ml) and chloramphenicol (34 μg/ml). Target protein expression was induced with the addition of IPTG (f/c 0.5 mM) when the bacterial culture OD_600nm_ increased to around 0.6, and was subsequently cultured for 1 h at 37 °C. Bacterial cells were then harvested and solubilized with Bugbuster (Sigma, St. Louis, MI) containing 250 U of Benzonaze (Sigma, St. Louis, MI), 30kU of recombinant Lysozyme (Fujifilm WAKO, Osaka, Japan) and 0.3% N-lauroyl sarcosine solution (Nakarai Tesque, Kyoto, Japan). The proteins were purified using His GraviTrap (Themo Fisher Scientific, Rochester, NY).

MPB83 was purified from a culture filtrate of recombinant *M. smegmatis* secreting 6xHistidine-tagged MPB83 (rMPB83-His) with the following procedure. DNA encoding MPB83 with addition of an NdeI site at the N terminal, 6XHistidine and a KpnI site at the C terminal was synthesized (Supplemental Table 1) and inserted into pSO246-AMI, which include an acetamidase promoter region at the site of NdeI and KpnI^50^. The complete plasmid (pSO246-AMI-mpb83) was electroporated into *M. smegmatis* under the conditions of 50 µF, 150 Ω, 1700 V with a 1.0 mm cuvette. Transformants were obtained by culturing on Middlebrook 7H10 agar (BD, Franklin Lakes, NJ) supplemented with 0.5% [v/v] glycerol and 10% ADC enrichment supplemented with 0.06% [v/v] oleic acid (7H11-OADC agar) containing 20 µg/ml of kanamycin for 4 days. Obtained colony was cultured in 7H9-ADC media containing 20 µg/ml of kanamycin, and expression of rMPB83-His was induced by addition of acetoamide at a final concentration of 0.2%. rMPB83-His expression in bacterial cells was confirmed by western blot using anti-HIS-direct monoclonal antibody (MEDICAL & BIOLOGICAL LABORATORIES, Nagoya, Japan). To obtain large scale rMPB83-His, Sauton media^51^ supplemented with 20 µg/ml kanamycin in a straight neck flask (Thermo Fisher Scientific, Waltham, MA) was utilized. Similarly, the expression of rMPB83-His was induced by addition of 0.2% (f/c) acetamide. The culture supernatant was filtered with 0.45 µm Rapid-Flow filter (Thermo Fisher Scientific, Waltham, MA) and subsequently purified with His GraviTrap. Purified MPB83, ESAT6, CFP10 and Ag85B were confirmed by SDS-PAGE with 15% polyacrylamide gel and silver staining method (Supplemental Fig. 1).

### Enzyme-linked immunosorbent assay for the detection of *Mtb* antigen-specific IgG

ELISA was performed in flat-bottomed, 96-well microtiter plates from Immulon1B (Thermo Fisher Scientific, Waltham, MA) or MaxiSorp (Thermo Fisher Scientific, Waltham, MA). One hundred µl of five µg/ml antigens in PBS were added to each well. The plate was incubated at 4 °C overnight. The wells were then washed 5 times with 300 µl PBS with 0.05% tween 20 (PBS-T). Three hundred µl of 5% skim milk in PBS-T was added and incubated at 4 °C overnight for blocking. The wells were washed 5 times with 300 µl PBS-T. Serum specimens were diluted 200 times with PBS-T containing 1% skim milk, and 100 µl of each was added to a well. The plates were then incubated at 37 °C for 1 h, and washed 5 times with 300 µl PBS-T. Rabbit antiserum against elephant IgG was provided by the University of Miyazaki center for animal disease control^52^. The antiserum was diluted 5,000 times for evaluating immunodominance (Fig. 3), or 10,000 times for validation of antibody specificity and longitudinal evaluation (Figs. 4 and 5); 100 µl was added to each well. The plates were again incubated at 37 °C for 1 h, and washed 5 times with 300 µl PBS-T. Donkey peroxidase-conjugated anti-rabbit IgG (H + L) antibody (Jackson ImmunoResearch Laboratories, Inc., West Grove, PA) was diluted 10,000 times for immunodominance evaluation, or 40,000 times for specificity validation and longitudinal evaluation;100 µl was added to each well. Sera and antibody dilutions were carried out with PBS-T containing 1% skim milk. The plates were incubated at 37 °C for 1 h and the wells washed 5 times with 300 µl PBS-T. One hundred µl of TMB 1-component microwell peroxidase substrate (SureBlue reserve, Seracare Life Sciences, Milford, MA) was added to each well. The plates were incubated at room temperature for 5 min with shading from light. One hundred µl of stop solution was added to each well to stop further color development. Abs level was measured at OD 450 nm. The IgG level was measured in duplicate for each sample, and the average was calculated. Furthermore, ELISA was repeated 3 times including 2 pilot tests for longitudinal evaluation (Fig. 5), and the representative data was presented.

### Statistics

For specificity validation of PPD and 3 *Mtb* antigens, IgG levels in 7 healthy elephants were compared using Tukey honestly significant difference test. Regarding the longitudinal IgG evaluation in the TB positive elephant, Wilcoxon signed-rank test was used to compare Ag85B-specific IgG values with those of the control.

### Ethical statement

All methods in this study were performed in accordance with relevant guidelines and regulations. All sera used in this study were collected by veterinarians in accordance with the Code of Ethics and Welfare of the Japanese Association of Zoos and Aquariums. The use of these sera was approved by each zoo that manages the elephants after full informed consent.

The sera were collected at the Fukuyama Zoo veterinary clinic, which is notified as a clinical facility for captive animals in accordance with Article 3 of the Veterinary Therapy, received by Hiroshima Prefecture (Notification ID. Fuku-3). The tests performed in this study were conducted as TB tests that were required for veterinary care.

Approval for animal experimentation was confirmed not to be required for this study by the Institutional Animal Care and Use Committee of Niigata University.

## Supplementary Information

Supplementary Information.
