# Supplementary material for: Monitoring IgG against Mycobacterium tuberculosis proteins in an Asian elephant cured of tuberculosis that developed from long-term latency
Source: Sci Rep. 2022 Mar 12;12:4310. doi: 10.1038/s41598-022-08228-7 (PMC8917326; doi:10.1038/s41598-022-08228-7)
Supplement: Supplementary file 1 — Supplementary Information. [file 41598_2022_8228_MOESM1_ESM.pdf]

Supplementary Information for

Monitoring IgG against *Mycobacterium tuberculosis* proteins in an Asian elephant cured of tuberculosis that developed from long-term latency

Satoshi Ishikawa[1,2*], Yuriko Ozeki[1], Satomi Suga[2], Yasuhiko Mukai[2], Haruka Kobayashi[1], Erina Inouchi[1], Shaban A. Kaboso[1], Gebremichal Gebretsadik[1], Desak Nyoman Surya Suameitria Dewi[1,3], Akihito Nishiyama[1], Yoshitaka Tateishi[1], Hayato Takihara[4], Shujiro Okuda[4,5], Shiomi Yoshida[6,7], Naoaki Misawa[8] and Sohkichi Matsumoto[1,9*]

Affiliations:
[1]Department of Bacteriology, Niigata University School of Medicine, 1-757, Asahimachi-Dori, Chuo-ku, Niigata, Niigata, 951-9510, Japan
[2]Fukuyama Zoo, 276-1, Fukuda, Ashida-cho, Fukuyama, Hiroshima, 720-1264, Japan
[3]Department of Microbiology, Faculty of Medicine, Universitas Ciputra, CitraLand CBD Boulevard, Made, Sambikerep, Surabaya, 60219, Indonesia
[4]Division of Bioinformatics, Niigata University Graduate School of Medical and Dental Sciences, 2-5274, Gakkocho-dori, Chuo-ku, Niigata, Niigata, 951-8514, Japan
[5]Medical AI Center, Niigata University School of Medicine, 2-5274, Gakkocho-dori, Chuo-ku, Niigata, Niigata, 951-8514, Japan
[6]Nagasaki University Graduate School of Biomedical Sciences, 1-12-4 Sakamoto, Nagasaki, 852-8523, Japan
[7]National Hospital Organization Kinki-chuo Chest Medical Center Clinical Research Center, 1180 Nagasone-cho, Kita-ku, Sakai, Osaka, 591-8555, Japan
[8]Graduate School of Medicine and Veterinary Medicine, University of Miyazaki, 5200, Kihara, Kiyotake-cho, Miyazaki, Miyazaki, 889-1692, Japan
[9]Laboratory of Tuberculosis, Institute of Tropical Disease, Universitas Airlangga, Kampus C Jl. Mulyorejo, Surabaya, 60115, Indonesia

***Corresponding author emails:**
SI, sat.ishikawa@kyf.biglobe.ne.jp , SM, sohkichi@med.niigata-u.ac.jp

Supplemental Table 1

CATATGATTA	ATGTTCAAGC	GAAACCGGCG	GCGGCGGCGA	GCCTGGCGGC
1	11	21	31	41
GATTGCGATT	GCGTTCCTGG	CGGGTTGCAG	CAGCACCAAA	CCGGTTAGCC
51	61	71	81	91
AGGACACCAG	CCCGAAGCCG	GCAACCAGCC	CGGCAGCACC	GGTTACCACC
101	111	121	131	141
GCGGCGATGG	CGGACCCGGC	AGCAGATCTG	ATTGGTCGTG	GCTGCGCACA
151	161	171	181	191
GTATGCAGCA	CAAAACCCGA	CCGGTCCGGG	CAGCGTGGCA	GGTATGGCAC
201	211	221	231	241
AAGACCCGGT	TGCAACCGCA	GCAAGCAACA	ACCCGATGCT	GAGCACCCTG
251	261	271	281	291
ACCAGCGCAC	TGAGCGGCAA	GCTGAACCCG	GACGTGAACC	TGGTTGATAC
301	311	321	331	341
CCTGAACGGT	GGCGAGTACA	CCGTGTTCGC	GCCGACCAAC	GCGGCGTTTG
351	361	371	381	391
ATAAACTGCC	GGCGGCGACC	ATTGACCAGC	TGAAGACCGA	TGCGAAACTA
401	411	421	431	441
CTGAGCAGCA	TCCTGACCTA	TCACGTTATT	GCAGGTCAAG	CGAGCCCGAG
451	461	471	481	491
CCGTATTGAT	GGCACCCACC	AGACCCTGCA	GGGTGCGGAT	CTGACCGTGA
501	511	521	531	541
TTGGTGCGCG	TGACGATCTG	ATGGTTAACA	ACGCGGGTCT	GGTGTGCGGT
551	561	571	581	591
GGCGTTCACA	CCGCGAACGC	GACCGTTTAC	ATGATTGACA	CCGTTCTGAT
601	611	621	631	641
GCCGCCGGCG	CAGCACCACC	ACCACCACCA	CTAATAGGTA	CC
651	661	671	681	691

The DNA sequence encoding MPB83 with addition of a NdeI site (blue underline, 1-6) at the N terminal, 6XHistidine (red underline, 664-681) and a KpnI site (blue underline, 587-692) at the C terminal

Supplemental Figure 1

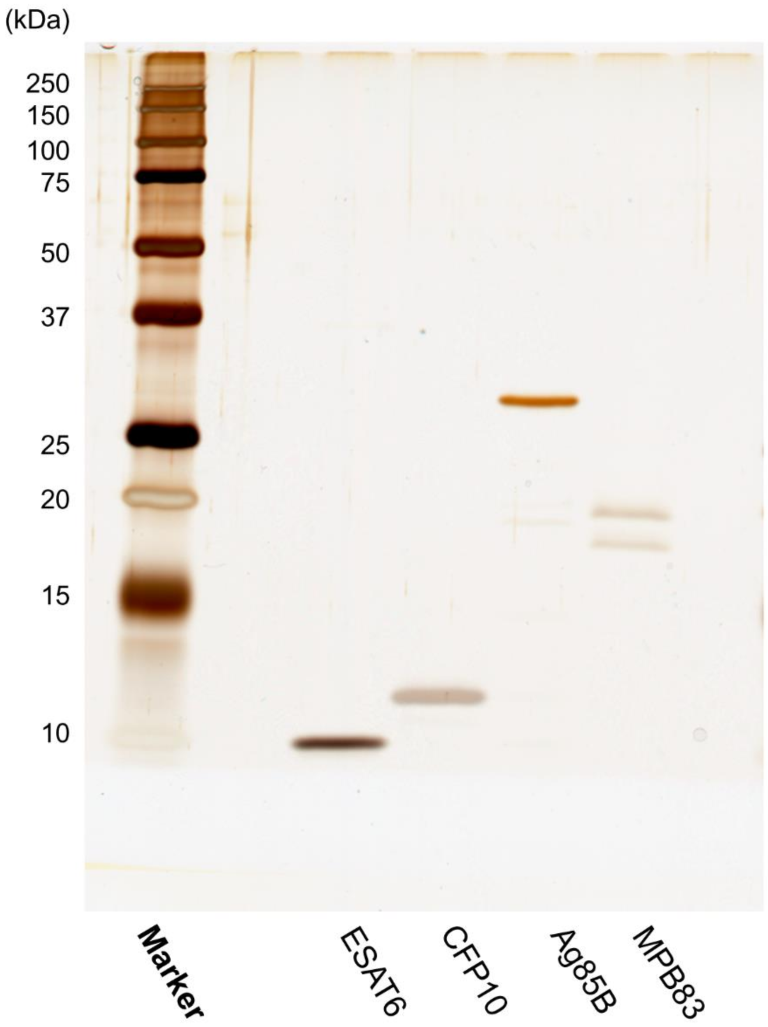

SDS-PAGE of purified proteins

Purified proteins were analyzed with SDS-PAGE with 15% polyacrylamide gel, followed to detect with silver stain.

Supplemental Figure 2

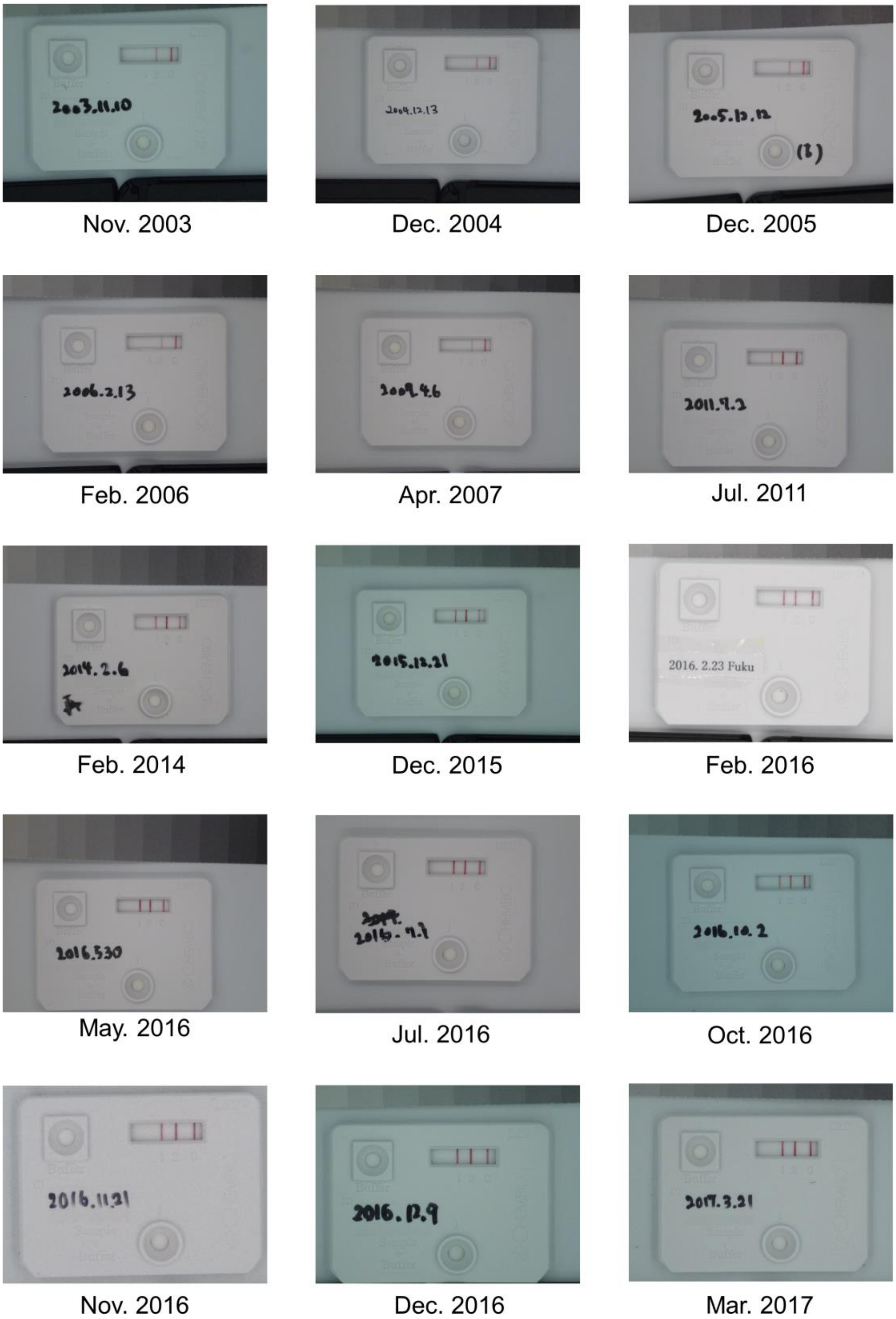

Nov. 2003 | Dec. 2004 | Dec. 2005

Feb. 2006 | Apr. 2007 | Jul. 2011

Feb. 2014 | Dec. 2015 | Feb. 2016

May. 2016 | Jul. 2016 | Oct. 2016

Nov. 2016 | Dec. 2016 | Mar. 2017

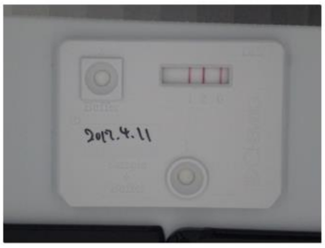

Apr. 2017

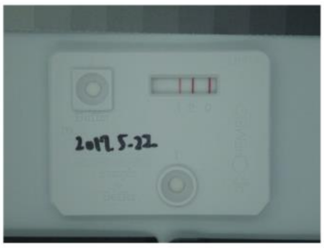

May. 2017

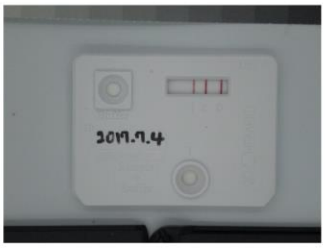

Jul. 2017

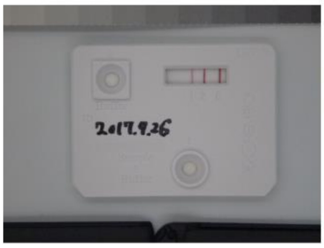

Sep. 2017

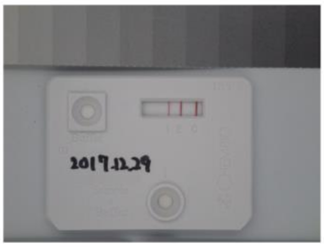

Dec. 2017

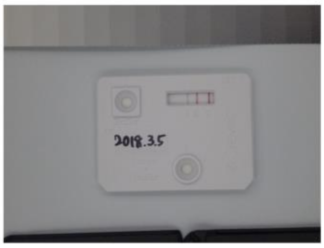

Mar. 2018

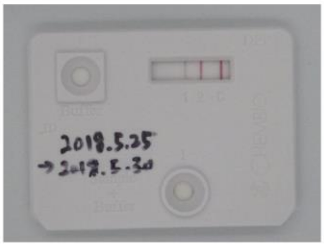

May. 2018

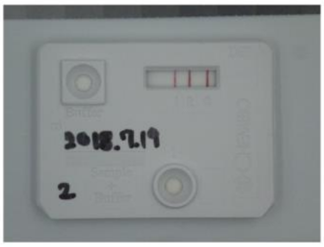

Jul. 2018

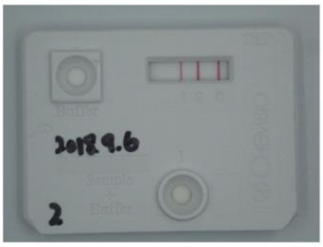

Sep. 2018

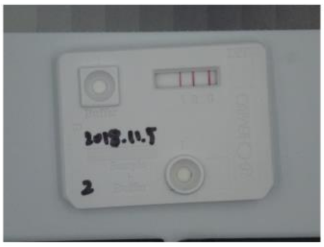

Nov. 2018

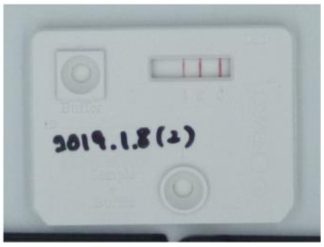

Jan. 2019

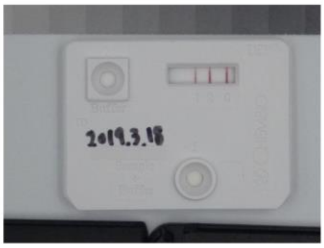

Mar. 2019

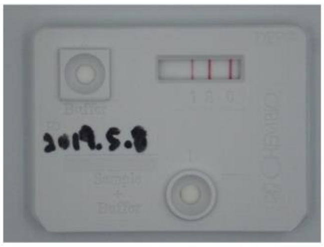

May. 2019

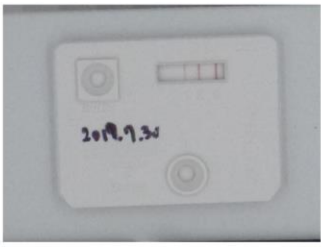

Jul. 2019

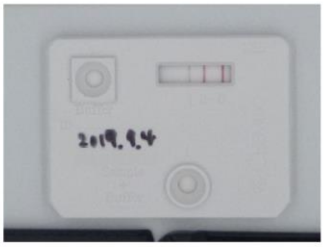

Sep. 2019

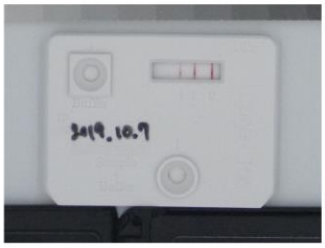

Oct. 2019

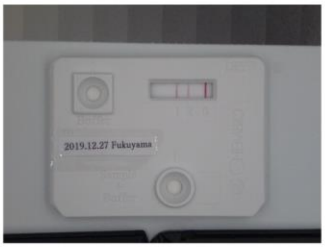

Dec. 2019

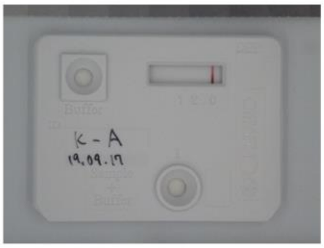

One of the healthy elephants

Uncropped images of DPP results with 32 sera from the TB positive elephant and one of the sera from the healthy elephants

Full images of DPP results in Figure 2 were presented as Supplemental Figure 2. Thirty-two images from Nov. 2003 to Dec. 2019 show the results of DPP performed with sera collected at different time points from the TB positive elephant. The last image with the words "One of the healthy elephants" is the same one as A-c in Figure 2.

In the test windows, the appearance of the red line on the left (T1 line) and the red line in the middle (T2 line) indicate the presence of MPB83 and ESAT6/CFP10 antibodies, respectively. The red line on the right is the control: if it does not appear, the test is invalid.
